# Oral Spirochete *Treponema denticola* Intraoral Infection Reveals Unique miR-133a, miR-486, miR-126-3p, miR-126-5p miRNA Expression Kinetics during Periodontitis

**DOI:** 10.3390/ijms241512105

**Published:** 2023-07-28

**Authors:** Chairmandurai Aravindraja, Syam Jeepipalli, Krishna Mukesh Vekariya, Ruben Botello-Escalante, Edward K. L. Chan, Lakshmyya Kesavalu

**Affiliations:** 1 Department of Periodontology, College of Dentistry, University of Florida, Gainesville, FL 32610, USA; aravindrchairman@ufl.edu (C.A.);; 2 Department of Oral Biology, College of Dentistry, University of Florida, Gainesville, FL 32610, USA

**Keywords:** periodontal disease, miRNAs, NanoString analysis, transient miRNA expression, *T. denticola*

## Abstract

miRNAs are major regulators of eukaryotic gene expression and host immunity, and play an important role in the inflammation-mediated pathways in periodontal disease (PD) pathogenesis. Expanding our previous observation with the global miRNA profiling using partial human mouth microbes, and lack of in vivo studies involving oral spirochete *Treponema denticola*-induced miRNAs, this study was designed to delineate the global miRNA expression kinetics during progression of periodontitis in mice infected with *T. denticola* by using NanoString nCounter^®^ miRNA panels. All of the *T. denticola*-infected male and female mice at 8 and 16 weeks demonstrated bacterial colonization (100%) on the gingival surface, and an increase in alveolar bone resorption (*p* < 0.0001). A total of 70 miRNAs with at least 1.0-fold differential expression/regulation (DE) (26 upregulated and 44 downregulated) were identified. nCounter miRNA expression profiling identified 13 upregulated miRNAs (e.g., miR-133a, miR-378) and 25 downregulated miRNAs (e.g., miR-375, miR-34b-5p) in *T. denticola*-infected mouse mandibles during 8 weeks of infection, whereas 13 upregulated miRNAs (e.g., miR-486, miR-126-5p) and 19 downregulated miRNAs (miR-2135, miR-142-3p) were observed during 16 weeks of infection. One miRNA (miR-126-5p) showed significant difference between 8 and 16 weeks of infection. Interestingly, miR-126-5p has been presented as a potential biomarker in patients with periodontitis and coronary artery disease. Among the upregulated miRNAs, miR-486, miR-126-3p, miR-126-5p, miR-378a-3p, miR-22-3p, miR-151a-3p, miR-423-5p, and miR-221 were reported in human gingival plaques and saliva samples from periodontitis and with diabetes. Kyoto Encyclopedia of Genes and Genomes (KEGG) analysis revealed various functional pathways of DE miRNAs, such as bacterial invasion of epithelial cells, Ras signaling, Fc gamma R-mediated phagocytosis, osteoclast differentiation, adherens signaling, and ubiquitin mediated proteolysis. This is the first study of DE miRNAs in mouse mandibles at different time-points of *T. denticola* infection; the combination of three specific miRNAs, miR-486, miR-126-3p, and miR-126-5p, may serve as an invasive biomarker of *T. denticola* in PD. These miRNAs may have a significant role in PD pathogenesis, and this research establishes a link between miRNA, periodontitis, and systemic diseases.

## 1. Introduction

The microRNAs (miRNAs, miR) are widely recognized as key regulators of various biological processes, and have physiological functions in modulating expression of many important genes [1]. In the last two decades, there has been significant progress in miRNA research, and miR have been linked to numerous human diseases (cardiovascular disease, HIV, diabetes, hypertension, cancer). Currently, more than 2000 miRNAs have been identified in humans, and they collectively regulate one-third of the genes in the genome [2]. Each specific miRNA can have hundreds of different targets that affect entire gene expression networks [3]. miRNAs have been implicated in many diseases, and regulate the immune response of hosts infected with bacteria such as *Treponema pallidum* [4], *Helicobacter pylori* [5], *Mycobacterium avium* [6], *M. tuberculosis* [7], *Salmonella* [8], and *Listeria monocytogenes* [9]. Hence, understanding the expression pattern of miRNAs could potentially lead to the development of novel diagnostic biomarkers for various complicated microbial infection-driven inflammatory diseases, including periodontal disease (PD). PD is a dysbiotic chronic inflammatory disease caused by microbes interacting in the human subgingival sulcus. Interaction of various bacteria in the human subgingival sulcus results in host abnormal immune response, specifically an inflammatory response leading to infectious gingivitis and periodontitis, characterized by resorption of the alveolar bone and tooth loss [10]. 

Several inflammatory miRNAs, such as miR-21 [11], miR-146a [12,13], miR-155 [14,15], and miR-132 [16], were reported to be involved in the initiation and progression of PD. However, none of these inflammatory miRNAs were differentially expressed (DE) in a recent report that deployed partial human mouth microbes (PAHMM) in an ecological time-sequential polybacterial infection mouse model (ETSPPI) [17]. This observation suggested that various miRNAs are involved in PD, and the miRNA expression pattern may be bacterium-specific and time-dependent. *T. denticola* is one of the red complex major spirochetes commonly colonized in the subgingival cavity and strongly implicated in PD [18]. Though it resides in the gingival sulcus, high proportions of *T. denticola* were observed in the subgingival space in patients with gingivitis and periodontitis [19,20]. Recent reports with periodontitis and gingivitis patients’ subgingival plaques revealed the presence of highly taxonomically diverse communities of treponemes [21]. The virulence factors (chymotrypsin-like protease, phospholipase C, oligopeptidase, and endopeptidase) from *T. denticola* can disrupt epithelial cells and stimulate host immune responses [19], and induce interleukin-36γ expression in human oral gingival keratinocytes [22]. After its stimulation, *T. denticola* can successfully activate the host innate immune response in a TLR2-dependent fashion [23], enter the circulatory system, and colonize in the distal organs [24,25]. We have reported the intracellular localization of viable *T. denticola* in gingiva, salivary glands, and the aorta. We have also reported its genomic DNA in the aortic tissues, and identified inflammatory mediators in the serum of mice after *T. denticola* infection [26]. Studies have identified the presence of *T. denticola* DNA in the atheromatous plaques of atherosclerotic patients [27,28]. *T. denticola* was detected in a metagenomic analysis of ancient human bone tissue biopsies from Otzi the Iceman, a 5300-year-old Copper Age natural ice mummy, giving credence to hematogenous dissemination of this periodontal spirochete [29]. *T. denticola* has also been closely linked with Alzheimer’s Disease (AD), and recent in vivo studies have shown the tau hyperphosphorylation by *T. denticola* [30]. Furthermore, this is the first study to use a high-throughput approach in exploring altered miRNA profiles from *T. denticola*-infected mouse gingival tissues at two time-points (8-weeks and 16-weeks) and sham-infected normal tissues.

To date, the role of miRNAs in response to *T. denticola* live infection in a mouse model of PD has not been explored. The aims of this study were to determine whether intraoral infection of mice with *T. denticola* could lead to the global alteration of miRNA expression patterns, and assess the link between miRNAs and *T. denticola*-induced gingival inflammation. A systematic investigation of the miRNA expression profiles in mouse mandibles of *T. denticola*-infected C57BL/6J mice during 8- and 16-weeks compared with uninfected mice was conducted using high-throughput NanoString nCounter^®^ miRNA expression panels. Gingival infection of *T. denticola* significantly increased ABR. We reported *T. denticola* colonization of the gingival surface on all of the mice, and a significant increase in ABR at 8-weeks and 16-weeks duration of infected mice. Unique miRNAs were identified in both 8-weeks and 16-weeks *T. denticola*-infected mice. Only one miRNA, miR-126-5p, was commonly expressed at both time-points. This study provides increased understanding of the specific miRNA signature of *T. denticola* in a PD mouse model. 

## 2. Results

### 2.1. Effective Colonization of T. denticola in Mouse Gingival Tissues

Oral gingival plaque samples from mice after *T. denticola* infection were used for 16S rRNA gene-specific colony PCR, and it demonstrated the presence of *T. denticola* 16S rRNA gene amplicon in agarose gel electrophoresis. After the first infection cycle, only one mouse from each of the 8-weeks and 16-weeks infected group was positive for *T. denticola* gDNA. However, all of the mice (100%) tested positive for *T. denticola* after the second infection cycle (Table 1). None of the sham-infected mice were positive at any point for genomic DNA of *T. denticola*. These results confirmed the colonization of *T. denticola* in both 8-weeks and 16-weeks *T. denticola*-infected groups. 

### 2.2. T. denticola Infection Increased Alveolar Bone Resorption with Minimal Dissemination of Treponemes to Distal Organs

**Figure 1:**
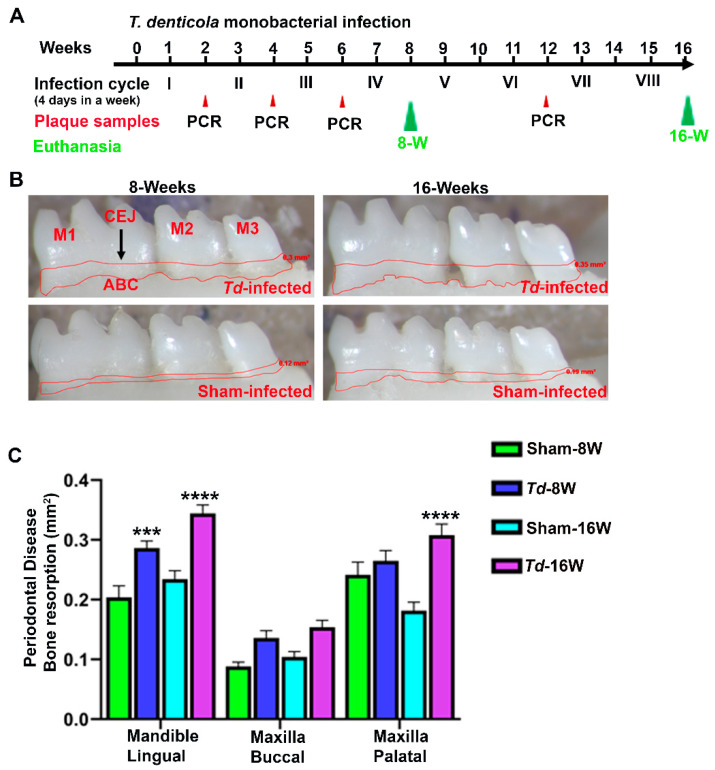
Gingival infection of *T. denticola* significantly induced ABR. (**A**). Schematic diagram of the experimental design depicting the monobacterial *T. denticola* infection (4 days per week on every alternate week), plaque sampling for PCR, and euthanasia. (**B**). Representative images showing horizontal ABR (mandible lingual view) of *T. denticola*-infected and sham-infected mice with the area of bone resorption outlined from the alveolar bone crest (ABC) to the cementoenamel junction (CEJ). (**C**). Morphometric analysis of the mandible and maxillary ABR in mice. A significant increase in ABR was observed in *T. denticola*-infected mice compared to sham-infected mice at both 8-weeks and 16-weeks infected mice (**** *p <* 0.0001; *** adjusted *p*-value = 0.0004; ordinary two-way ANOVA). Data points and error bars are mean ± SEM (*n* = 10).

The periodontal disease outcome of the bacterial infection was examined using bone morphometry by measuring the horizontal ABR. Mice at both 8-weeks and 16-weeks infection with *T. denticola* showed significantly higher ABR in the mandible (lingual) (adjusted *p-value =* 0.0004 for 8-weeks group; *p* < 0.0001 for 16-weeks group) (Figure 1B,C). Similarly, significantly higher ABR was observed in the maxilla (palatal) (*p <* 0.0001 for 16-weeks *T. denticola*-infected mice). No IgG antibody response was observed against *T. denticola* in both 8-weeks and 16-weeks infected mice. A total of 40% of the female mice infected with *T. denticola* at 16-weeks demonstrated bacterial dissemination in their lung tissues (Table 2). Robust bacterial dissemination to other distal organs such as the heart, brain, liver, kidney, and spleen was not observed.

### 2.3. NanoString Analysis of miRNAs in T. denticola-Infected Mandibles

**Figure 2:**
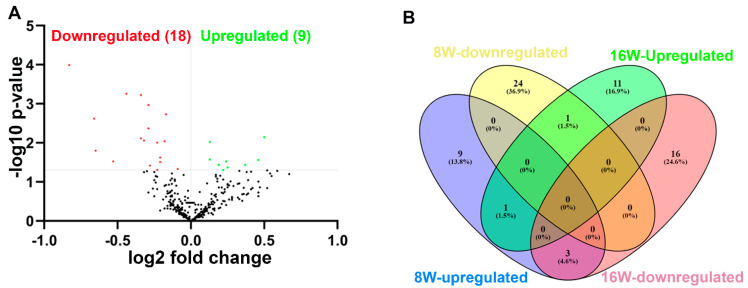
Differentially expressed (DE) miRNAs in *T. denticola*-infected mouse mandibles (8- and 16-weeks). (**A**). The volcano plot depicts the upregulated (green) and downregulated (red) miRNAs that showed a fold difference of ±1.1 with *p*-value of <0.05. The log2 fold change (FC) is on the *x*-axis, and the negative log of the *p*-value is on the *y*-axis. The black dots represent the miRNAs that do not pass the filter parameters. Nine significant upregulated miRNAs and eighteen downregulated miRNAs were identified in 16-weeks *T. denticola*-infected mice (*n* = 10). (**B**). Venn diagram analysis illustrates the distribution of DE miRNAs in 8-weeks and 16-weeks infection with *T. denticola*. This analysis shows that only one miRNA, miRNA-126-5p. was commonly upregulated in both 8- and 16-weeks infected mice. All other upregulated and downregulated miRNAs were unique to the 8- and 16-weeks infected mice.

Global miRNA profiling was carried out in the mandibles of mice infected with *T. denticola* at 8-weeks and 16-weeks infection. A *p*-value of >0.05 and fold change (FC) of 1.1 was taken for analysis and considered to be significant. A total of 70 differentially regulated miRNAs were identified. nCounter miRNA expression profiling showed 13 upregulated miRNAs (e.g., miR-133a, miR-378) and 25 downregulated miRNAs (e.g., miR-375, miR-34b-5p) in *T. denticola* infected mouse mandibles compared to sham-infected mouse mandibles during 8 weeks of infection. Thirteen upregulated miRNAs (e.g., miR-486, miR-126-5p) and 19 downregulated miRNAs (miR-2135, miR-142-3p) were identified in *T. denticola*-infected mice compared to sham-infected mice during 16 weeks of infection (Table 3; Supplementary Tables S1–S3). Volcano plot analysis identified 9 upregulated and 18 downregulated miRNAs in the 8-weeks *T. denticola*-infected group (Figure 2A). miR-126-5p was the only one commonly upregulated miRNA in 8- and 16-weeks *T. denticola*-infected mouse mandibles, and all of the DE miRNAs were unique to the specific time-point (Figure 2B). Inflammatory miR-132 (FC 1.21; *p*-value: 0.01) was upregulated in 16-weeks infected mouse mandibles, but it was not upregulated in 8-weeks infected mouse mandibles. Other previously identified inflammatory miRNAs, such as miR-146a, miR-132, and miR-155, were not differentially expressed at both time-points.

### 2.4. Functional Pathway Analysis of Differentially Expressed miRNAs

**Figure 3:**
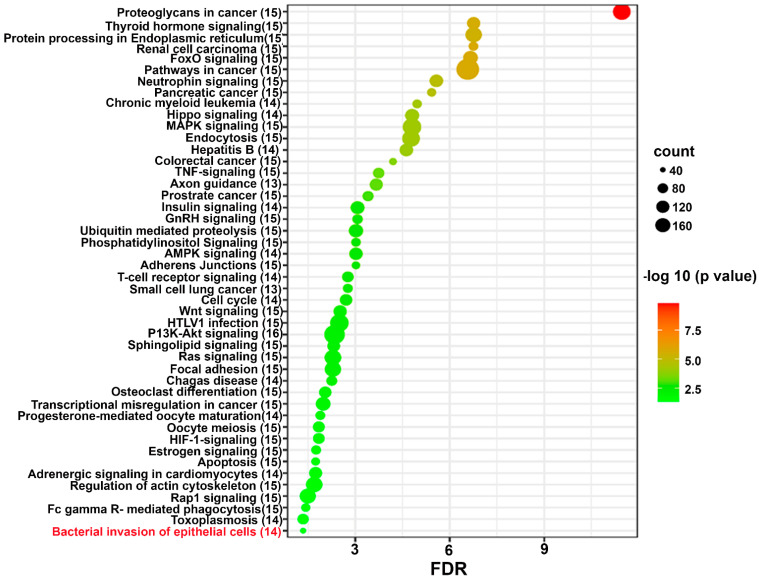
Predicted functional pathway analysis of DE miRNAs from *T. denticola*-infected mouse mandibles. Bubble plot of KEGG analysis on predicted target genes of DE miRNAs in *T. denticola*-infected mice compared to sham-infected mice. The KEGG pathways are displayed on the y-axis, and the x-axis represents the false discovery rate (FDR), which means the probability of false positives in all tests. The size and color of dots represent the number of predicted genes and corresponding *p*-value, respectively. Fourteen DE miRs were shown to be involved in bacterial invasion of epithelial cells.

**Figure 4:**
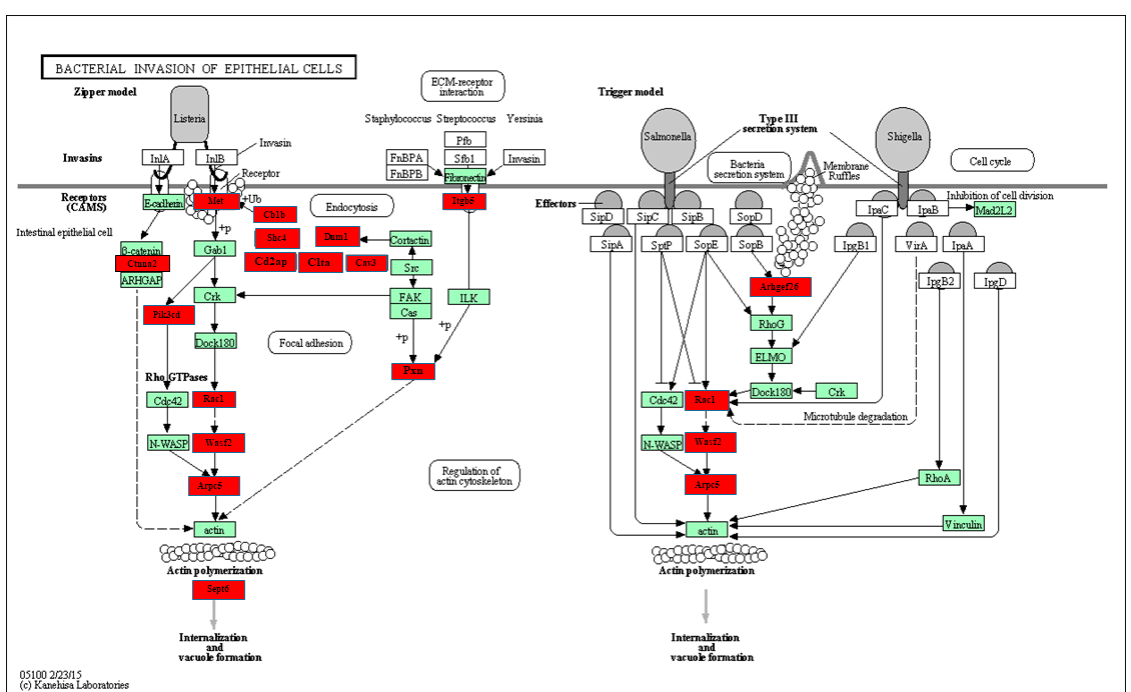
Significantly differentially expressed genes (identified by KEGG) involved in the bacterial invasion of epithelial cell signaling pathways. Red boxes indicate significantly increased expression based on miRNA profiles from NanoString analysis. Green boxes indicates no change in gene expression. Many pathogenic bacteria can invade phagocytic and nonphagocytic cells and colonize them intracellularly, then become disseminated to other cells. Invasive bacteria induce their own uptake by nonphagocytic host cells (e.g., epithelial cells) using two mechanisms, referred to as the zipper model and trigger model. *Listeria*, *Staphylococcus*, *Streptococcus*, and *Yersinia* are examples of bacteria that can enter using the zipper model. These bacteria express proteins on their surfaces that interact with cellular receptors, initiating signaling cascades that result in close apposition of the cellular membrane around the entering bacteria. *Shigella* and *Salmonella* are examples of bacteria entering cells using the trigger model.

Predicted functional pathway analysis of the DE miRNAs in both 8-weeks and 16-weeks *T. denticola*-infected mice identified several pathways (DIANA-miRPath): Hippo signaling, mitogen-activated protein kinase (MAPK) signaling pathway, Wnt (Wingless and Int-1) signaling pathway, and pathways related to various cancers, such as pancreatic cancer, small cell lung cancer, and prostate cancer. Further, 14 significantly DE miRNAs expressed in 8-weeks and 16-weeks *T. denticola*-infected mice were found to be involved in bacterial invasion of epithelial cells in signaling pathways (Figure 3 and Figure 4). Similarly, DE genes involved with the TNF signaling pathway were associated with leucocyte recruitment and activation, and increased inflammatory cytokine expression genes (Supplementary Figure S1). In addition, other pathways that are linked with infection and host cell associations, such as adherens junctions, endocytosis, TNF signaling, and apoptosis, were identified. A literature review of the identified upregulated miRNAs in 8-weeks and 16-weeks *T. denticola*-infected mice revealed that the commonly expressed miRNA-126-5p at both time-points was shown to be overexpressed in ovarian cancer cells [31], endothelial proliferation, atherosclerosis [32], and in ischemic stroke [33]. Another inflammatory miRNA, miR-132, was shown to inhibit differentiation of periodontal ligament cells into osteoblasts [34]. Detailed reported functions of the upregulated miRNAs are shown in Table 4. The number of target genes for each upregulated miRNA in 8- and 16-weeks of infection was analyzed using miRTarBase. We used mmu-miR-133 an example for an upregulated DE miRNA during 8 weeks of infection in identifying the target genes. *T. denticola*-infection induced mmu-miR-133, which has 28 different target genes with 28 different MiRTarBase IDs, as shown in Supplementary Table S4. Similarly, we used mmu-miR-486 an example for upregulation during 16 weeks of infection in identifying the target genes. *T. denticola* infection induced mmu-miR-486, which has 41 different target genes with 41 different MiRTarBase IDs, as shown in Supplementary Table S5. 

## 3. Discussion

Recent studies using partial human mouth microbes (PAHMM) in an ecological time-sequential polybacterial periodontal infection model (ETSPPI) showed sex-specific differential miRNA expression during PD [17]. This PD mouse model utilized five different bacteria, such as *Streptococcus gordonii* (early colonizer), *Fusobacterium nucleatum* (intermediate colonizer), *Porphyromonas gingivalis*, *Treponema denticola,* and *Tannerella forsythia* (late colonizers). We have developed highly efficient five multispecies infection-induced miRNA expression, but the role of DE miRNAs on monobacterial infection has not been examined to better understand the induction process of individual bacterium contribution to periodontal disease. Hence, we designed this study to analyze the global miRNA expression patterns at two different time-points in *T. denticola*-induced PD. We analyzed *T. denticola* colonization on the mouse gingival surface, horizontal ABR measurements, intravascular dissemination of *T. denticola* to distal organs, and global miRNA profiling in *T. denticola* mice infected at 8- and 16-weeks. All of the mice infected with *T. denticola* at both time-points showed 100% bacterial colonization on the gingival surface, which was confirmed via 16S rRNA gene amplification. Significantly higher ABR was also observed in *T. denticola*-infected mice at both time-points. These data were in line with our previously published data, where significantly higher ABR was observed in TgCRND8 mice and ApoE^−/−^ mice infected with *T. denticola* through gingival infection [69]. Similarly, the observed ABR data were also consistent with our recent study using a PAHMM-infected ETSPPI mouse model [17]. Based on the observed significant ABR in both 8- and 16-weeks infection, we once again confirmed that *T. denticola* is one of the major periodontal bacteria in the progression of PD.

miRNA profiling of mouse mandibles infected with *T. denticola* identified unique miRNA gene signatures with both 8- and 16-weeks infection. Most of the DE miRNAs were unique and specific to each time-point, and confirmed that miRNA expression is transient and time-dependent. The DE miRNAs in 8-weeks infected mice were not found in the 16-weeks infected mice. Though all of the DE miRNAs were unique, miR-126-5p was commonly upregulated at both time-points. It is interesting that miR-126-5p has been observed in gingival tissues in periodontitis patients [12,15]. Further, miR-126-5p has been shown as a potential biomarker in patients with periodontitis and coronary artery disease [28]. miRNA-126-5p has been found to play a dual role in the atherosclerotic pathway, in which it prevents plaque formation by downregulating VCAM-1 expression, and augments the atherosclerotic process through muscle cell proliferation and activation [28]. In addition, miR-126-5p plays a major role in ovarian cancer by promoting chemoresistance of ovarian cancer cells [31], and attenuates the blood–brain barrier after ischemic stroke [33]. As miR-126-5p was upregulated at both time-points, it may be considered as a potential biomarker for PD.

Among the 13 upregulated miRNAs (e.g., miR-133a (1.83 FC); miR-378 (1.57 FC); miR-22 (1.55 FC); miR-151-5p (1.15 FC)) in the 8-weeks *T. denticola*-infected mice, three human homologs of miRNAs (hsa-miR-378a-3p, hsa-miR-22-3p, hsa-miR-151a-3p) were shown to be observed in unstimulated saliva in patients with generalized chronic periodontitis [70]. Similarly, among the 13 upregulated miRNAs in 16-weeks *T. denticola*-infected mice, five miRNAs, viz., miR-486, miR-126-3p, miR-126-5p, miR-423-5p, and miR-221, were reported in human gingival plaques samples with periodontitis, saliva of patients with periodontitis and diabetes [12,15,43,57,71], and in unstimulated saliva samples in patients with generalized chronic periodontitis [70]. miR-133 has therapeutic application in heart disease as a key regulator of cardiac hypertrophy [72], a protective effect against myocardial ischemia-reperfusion (IR) injury in Sprague Dawley rats, [73] and improves cardiac function and fibrosis via inhibiting Akt in heart failure rats [74]. miR-486 was also reported to promote cardiac angiogenesis through the fibroblastic MMP-19-VEGFA cleavage signaling pathway [54]. *Treponema* species possess lipopolysaccharides (LPS) on their outer membrane [75], and elevated miR-486-5p was observed during acute lung injury in human subjects and LPS-induced acute lung injury in mice [76]. Elevated miR-486-5p has been reported to prevent endothelial dysfunction through anti-inflammation and antioxidant mechanisms [77]. miR-126-3p was significantly overexpressed in gingival tissues of chronic and aggressive periodontitis. miR-126 is also associated with other inflammatory disease conditions such as obesity and arthritis [78]. Since these three miRNAs were observed in both human periodontal gingival tissues and *T. denticola*-induced experimental periodontitis, further in-depth analysis is needed to consider miR-486, miR-126-3p, and miR-126-5p as potential biomarkers for periodontal disease.

Another inflammatory miR-132 was also upregulated during 16-weeks *T. denticola* infection. We have previously shown that THP-1 cells infected with live and heat-killed *T. denticola* did not significantly increase miR-132 expression. Thus, the observed miR-132 expression under in vivo conditions suggests that miR-132 expression may vary under in vitro and in vivo conditions, and further validation needs to be performed to confirm this observation [16]. Other widely reported inflammatory miRNAs, miR-155 and miR-146a, were not upregulated in both 8- and 16-weeks *T. denticola*-infected mice. Other upregulated miRNAs such as miR-378, miR-22, miR-451, miR-496, miR-151-5p, miR-101a, miR-423-5p, and miR-221 were found to be closely associated with various human cancers. miR-378 plays a major role in hepatic inflammation and fibrosis by targeting the NF*κ*B–TNFα axis [36]. miR-22 promotes cell differentiation, tumor initiation, progression, and metastasis by maintaining Wnt/β-catenin signaling and cancer stem cell function [38]. miR-451 plays an important role in lung tumor progression, and is highly dysregulated in lung cancer [42]. miR-496 mediates tumor metastasis in colorectal cancer through Wnt signaling pathways [46]. Further, miR-423-5p was noted to be overexpressed in breast cancer, causing severe invasion of tumor cells through the NF*κ*B signaling pathway [62]. Thus, most of the upregulated miRNAs during *T. denticola* infection may be involved in tumor progression and metastasis by mediating the NF*κ*B pathway, TNF signaling, or Wnt signaling pathways. It is obvious that the upregulated miRNAs in the current study may have these functions, as they are involved in inflammation-mediated periodontal infection, which leads to NF*κ*B activation for transcription of inflammatory genes.

Several reports have identified a link between *T. denticola* and various brain disorders such as AD and stroke [79,80,81,82]. Interestingly, the upregulated miR-101b was found to be an important mediator of tauopathy and dendritic abnormalities in AD progression [63]. Further, miR-136 was identified as a potential biomarker for human mild traumatic brain injury [40]. These reports strongly suggest a possible link between bacteria-induced gingival and cerebral inflammation via miR-101b and miR-136, respectively. The other upregulated miR-30a and -30c were observed to be highly expressed in gingival tissues in periodontitis patients, and play major roles in the inhibition of osteogenesis followed by periodontitis [44,45]. 

Predicted functional analysis pathways using KEGG revealed interesting findings. Most of the pathways targeted by the miRNAs that were differentially expressed during *T. denticola* infection were linked to bacterial pathogen recognition and clearance, including endocytosis, bacterial invasion of cells, and FcR-mediated phagocytosis. Further, 14 miRNAs were found to be closely associated with bacterial invasion of epithelial cells. This is the first study that identified the specific miRNAs involved in *T. denticola* invasion of epithelial cells that could be potentially used as biomarkers for periodontitis. Other pathways linked to infection and host cell associations such as adherens junctions, endocytosis, TNF signaling, lysosome, and TGF-β signaling pathways were also identified. A recent clinical study identified functional circRNAs, and predicted a circRNA–miRNA–mRNA regulatory network in periodontitis [83]. To conclude, this is the first study to report the global miRNA expression kinetics of *T. denticola* infection. Our data show that monoinfection with the red complex bacterium *T. denticola* can induce experimental PD, and we believe that red complex bacteria are important in human PD. However, how PD is initiated by components of the red complex is not completely understood. There are many questions that remained unanswered, and the role that *T. denticola* can play alone in the induced PD in experimental systems cannot be discounted. In addition, *T. denticola* induced several DE miRNAs, and some of those miRNAs are also observed in human gingival and saliva samples of patients with periodontitis, and periodontitis patients with diabetes. This study highlights the transient expression of miRNA in mouse gingival tissues, and their expression pattern varies based on the length of *T. denticola* infection. Further, in-depth analysis of the identified DE miRNAs using KO mouse models will provide additional details on PD pathogenesis and its link with other systemic diseases.

## 4. Materials and Methods

### 4.1. Gingival Infection of T. denticola to Induce Periodontitis in C57BL/6J Mouse

*T. denticola* ATCC 35405 (*Td*) was grown in GM-1 broth in an anaerobic growth chamber at 37 °C for three days. The log phase culture of *T. denticola* was harvested after centrifugation at 8000 rpm for 10 min, followed by washing with sterile 5 mL phosphate buffered saline (PBS). The pellets were resuspended in equal volumes of 3% carboxymethylcellulose and reduced transport fluid (RTF). A volume of 100 µL of bacterial suspension was taken for gingival infection. Both male and female mice were divided into four groups (*n* = 10) (Group-I: *Td*-infected-8 weeks; Group-II: *Td*-infected-16 weeks; Group-III: sham-infected-8 weeks; Group-IV: sham-infected-16 weeks). Bacterial infection was performed in each group of mice for 8- and 16-weeks (infection cycle consists of four days per week for every alternative week to induce chronic periodontitis) (Figure 1A). An equal volume of RTF and CMC was used as a vehicle control for sham-infected mice as described previously [17,26,69]. Kanamycin (500 mg/mL) was administered in sterile drinking water for three days to suppress the existing mouse oral bacteria, followed by rinsing with 0.12% chlorhexidine gluconate. After the antibiotic washout period, the mice were given a topical gingival infection of *T. denticola* (10^8^ cells each) [17,69,84,85]. Mice were euthanized after 8-weeks and 16-weeks of infections. Left mandibles and maxilla were stored in RNA*later* (Invitrogen, Waltham, MA, USA) until total RNA extraction, and all other distal organs such as brain, heart, lung, spleen, liver, and kidney were also collected. Right maxilla and mandibles were collected for bone morphometry measurements. Blood was collected through cardiac puncture, and the serum was stored at −20 °C for serum IgG analysis. All of the animal procedures were performed according to the guidelines framed by the University of Florida Institutional Animal Care and Use Committee (IACUC protocol number 202200000223).

### 4.2. T. denticola 16S rRNA Gene Amplification in Oral Plaques

Gingival plaque samples from the mice infected with *T. denticola* and sham infection were collected after four continuous days of infection using sterile cotton swabs. *T. denticola* gDNA was detected using 16S rRNA gene species-specific primer of the bacteria using Phusion High Fidelity Master Mix from New England Biolabs (NEB, Ipswich, MA, USA) as described previously [86,87,88,89]. Briefly, colony PCR was performed with a Bio-Rad Thermal Cycler using *T. denticola*-specific 16S rRNA gene-specific forward primer 5′-TAATACCGAATGTGCTCATTTACAT-3′, and reverse primer 5′-CTGCCATATCTCTATGTCATTGCTCTT-3′. Genomic DNA extracted from *T. denticola* was used as a template for positive control, and samples with no bacterial DNA were used as a negative control. PCR products were run on 1% agarose gel electrophoresis and visualized under UVP GelStudio touch Imaging System (Analytik Jena US LLC, CA, USA) [17,69]. 

### 4.3. Bacterial Systemic Dissemination to Distal Organs

Genomic DNA from an aliquot of the distal organs such as heart, lungs, liver, brain, kidney, and spleen from 16-weeks infected mice and sham-infected mice were extracted following a standard procedure described in the Qiagen Dneasy Blood and Tissue kit (Qiagen, Germantown, MD, USA). *T. denticola*-specific 16S rRNA gene-specific PCR was performed to confirm the presence of *T. denticola* gDNA in the distal organs [86,87,88,89].

### 4.4. Horizontal Alveolar Bone Resorption by Morphometry

The horizontal ABR area of *T. denticola*-infected and sham-infected mice was measured by histomorphometry, as described previously [86,87,88,89]. The right mandibles and maxilla of the infected and sham-infected mice were autoclaved for 20 min. The autoclaved mandibles and maxilla were defleshed, followed by incubation in 3% hydrogen peroxide for 30 min and air dried. Two-dimensional mandibles and maxilla molar teeth images were captured using a stereo dissecting microscope (Stereo Discovery V8, Carl Zeiss Microimaging, Inc, Thornwood, NY, USA). The area between the cemento–enamel junction to the alveolar bone crest of the buccal and palatal surfaces of the maxillary jaws and lingual mandibular jaw was measured by using the line tool (AxioVision LE 29A software version 4.6.3, Thornwood, NY, USA). Two examiners blinded to the *T. denticola* mice groups measured the ABR [17,26,69,90]. 

### 4.5. nCounter^®^ miRNA Expression Profiling Using NanoString Analysis

Mandibles (left; *n* = 10) from each mouse (four groups) were taken for NanoString analysis. The total RNA from each mandible was extracted based on the protocol described in the mirVana miRNA isolation kit (Ambion, Austin, TX, USA) and as described in our recent publications [91]. Briefly, mandibles from each mouse were homogenized using the handheld rotor–stator homogenizer with sterile individual TissueRuptor disposable probes (Qiagen; Germantown, MD, USA) for each specimen. After homogenization, each homogenized tissue was lysed in a denaturing lysis solution that stabilized RNAs and inactivated RNases. The lysed tissue samples were subjected to an acid-phenol:chloroform extraction that removed all the cellular components (protein, DNA, and other cellular products). The aqueous phase was removed, and 1.25 volume of 100% ethanol was added. This was transferred to the filter cartridge placed into the collection tube, and centrifuged for 15 s at 10,000× *g*. This cartridge was washed with wash solution-1 and wash solution-2/3. The total RNA was eluted from the filter cartridge with nuclease-free water. The RNA concentration and purity were assessed using a Take3 microvolume plate in an Epoch Microplate Spectrophotometer (BioTek, USA, Winooski, VT, USA), and RNA quantification was performed in technical duplicates for each specimen. 

The high-throughput nCounter^®^ miRNA Expression Panel (NanoString Technologies, Seattle, WA, USA) was used to study the DE of miRNA in 8-weeks and 16-weeks infected and sham-infected male and female mice, as detailed in [91]. The NanoString nCounter^®^ panel can identify 577 miRNAs in any RNA specimen, and by using molecular barcodes, NanoString can detect even a small number of miRNAs without the need for reverse transcription or amplification. Mouse miRNA expression profiling was performed using the NanoString nCounter^®^ Mouse miRNA Assay kit v1.5 (NanoString Technologies). This assay is a highly sensitive multiplexed method that detects miRNAs using molecular barcodes called nCounter reporter probes, without the need for reverse transcription. Specimen preparation involving annealing, ligation, and purification were performed based on the experimental procedure described in the nCounter^®^ miRNA assay panel kit, and as described in our recent report [91]. Briefly, the annealing master mix was prepared by combining annealing buffer, nCounter miRNA Tag reagent, and diluted miRNA assay controls. Further, the annealing master mix was aliquoted into each tube of the strip, and 100 ng of the total RNA from ten mandibles from each group was added to the respective tubes. The strip tube was transferred to the thermal cycler with the following conditions: 94 °C for 1 min, 65 °C for 1 min, 45 °C for 1 min, and 48 °C for hold.

Following annealing, ligation master mix (polyethylene glycol (PEG) and ligation buffer) was added to the strip tube. The strip tube was incubated, followed by the addition of ligase into the tube. The ligation was performed with the following conditions: 48 °C for 3 min, 47 °C for 3 min, 46 °C for 3 min, 45 °C for 3 min, 65 °C for 10 min, and 4 °C for hold. To separate the unligated tags, a purification step was performed after adding ligation cleanup enzyme and incubating at 37 °C for 1 h, 70 °C for 10 min, and 4 °C for holding. The RNase-free water was added to the strip tube, and the specimen was ready for hybridization with the nCounter reporter and capture probes. After denaturation, an aliquot from the miRNA sample preparation tube was taken along with the miRNA reporter code, hybridization buffer, and miRNA capture probe. The strip tubes were incubated in the thermal cycler, and the specimens were immediately processed for posthybridization with the nCounter analysis system at the Molecular Pathology Core at the University of Florida [91]. The nCounter^®^ Mouse miRNA Assay kit v1.5 provided six positive hybridization controls and eight negative control probes to monitor hybridization efficiency. All components and reagents needed for specimen preparation at the preparation station were taken from the nCounter master kit. Twelve specimens per cartridge were processed in a single run and followed by digital analysis, which involved the transfer of the cartridge to the multichannel epifluorescence digital analyzer. A cartridge definition file with a maximum field of view (FOV) count of 555 per flow cell was taken for digital analysis. The number of images taken per scan corresponded to the number of immobilized reporter probes on the cartridge. A separate reporter code count (RCC) file for each sample containing the count for each probe was downloaded and used for miRNA data analysis [17]. 

### 4.6. NanoString Data Analysis

Initial data analysis was performed using nSolver 4.0 as described previously [91]. After importing the RCC files to nSolver, stringent quality measures were taken as per the system recommendations. All 40 specimens passed the QC, and no flag lanes were observed. Raw data were generated after passing the QC. To reduce the background signal/noise, the background threshold count value was set as 52, and was calculated by taking an average of eight negative control probe counts from all 40 samples. miRNA gene normalization was performed based on the top 100 miRNA genes expressed. The normalized factor was calculated based on the geometric mean values of the miRNAs expressed in each specimen. All of the normalized data were analyzed further using ROSALIND (https://rosalind.bio/30, accessed on 7 March 2023), with a HyperScale architecture developed by ROSALIND, Inc. (San Diego, CA, USA) [92]. Fold changes in the genes were calculated based on the ratio of the difference in the means of the log-transformed normalized data to the square root of the sum of the variances of the specimens in the groups. For KEGG (Kyoto Encyclopedia of Genes and Genomes) pathway analysis, we analyzed the DE miRNAs in 8- and 16-weeks bacterial-infected mouse mandibles in the DIANA-miRPath v.3.0 database [93]. All of the DE miRNAs were entered using the MIMAT accession number in the DIANA-miRPath v3.0 database, with the threshold values of *p* < 0.05 and false discovery rate (FDR) correction applied to obtain unbiased empirical distribution, bacterial invasion of epithelial cell pathway, and TNF signaling pathway. Additionally, we analyzed the DE miRNAs in 8- and 16-weeks bacterial-infected mouse mandibles in miRTarBase, which is the experimentally validated microRNA–target interactions database (miRTarbase update 2022: an informative resource for experimentally validated miRNA–target interactions) [94]. A Venn diagram for upregulated and downregulated miRNAs in 8- and 16-weeks infection groups was drawn using Venny 2.1 as described previously [91].

### 4.7. Statistical Analysis

An ordinary two-way ANOVA with Tukey’s multiple comparisons was performed for multiple group comparison to determine the statistical significance using the statistical software Prism 9.4.1 (GraphPad Software, San Diego, CA, USA). All of the data in graphs are presented as mean ± SEM. Two-tailed t-testing was performed on the log-transformed normalized data that assumed unequal variance to identify the differential gene expression as described previously [91]. A *p*-value of <0.05 was considered to be statistically significant. 

## 5. Conclusions

This is the first in vivo oral spirochete *T. denticola* monobacterial infection study that reports the altered global miRNA kinetics in experimental periodontitis. This study found that the elevated miR-133a (1.8 FC) expression in mouse mandibles during 8 weeks of infection may play an important function in the initial immune response to *T. denticola* infection. Unique miRNAs were identified in both 8- and 16-weeks *T. denticola* infection. One miRNA (miR-126-5p) showed significant difference between 8- and 16-weeks infection and controls. It is interesting to note that miR-126-5p has been shown as a potential biomarker in patients with periodontitis and coronary artery disease. KEGG pathway analysis of the DE miRNAs revealed various functional pathways. Our study provides strong insights on the specific miRNA signature (periodontitis biomarkers) of *T. denticola* in a PD mouse model at two different time-points. However, reservations must be made before transposing this model to the complexity of periodontal disease in humans. 

## Figures and Tables

**Table 1:** Gingival plaque samples positive for *T. denticola* gDNA by PCR.

Group/Bacteria/Weeks	Positive Gingival Plaque Samples (*n* = 10)			
	2 weeks	4 weeks	6 weeks	12 weeks
Group I/*T. denticola* ATCC 35405 [8 weeks]	1/10	10/10	NC	---
Group II/*T. denticola* ATCC 35405 [16 weeks]	1/10	10/10	NC	10/10
Group III/Sham-infection [8 weeks]	0/10	NC	NC	---
Group IV/Sham-infection [16 weeks]	0/10	NC	NC	0/10

Total numbers of gingival plaque samples that were collected after infection (2, 4, 6, and 12 weeks) following *T. denticola* gingival infections and were positive as determined by PCR analysis. NC—not collected, to allow bacterial biofilm to adhere to gingival surface, invade epithelial cells, and multiply. The first value corresponds to the number of mice that tested positive for bacterial genomic DNA, and the second value corresponds to the total number of mice in the group.

**Table 2:** Distribution of *T. denticola* genomic DNA of periodontal bacteria to distal organs.

Positive Systemic Tissue Specimens for *T. denticola* (16-Weeks) (*n* = 5 Males and 5 Females)						
Bacterial Infection	Sex (M/F)	Heart	Lungs	Kidney	Liver	Spleen
*T. denticola*	M	0	0	0	0	0
	F	0	2	0	0	0

To analyze the bacterial systemic infection, total genomic DNA from an aliquot of the mouse heart (comprising the right atrium and right ventricle), lungs, kidney, liver, and spleen was extracted. The extracted genomic DNA was examined for the presence of bacterial DNA through the *T. denticola*-specific 16S rRNA gene primers. A total of 40% of the female mice infected with *T. denticola* at 16-weeks demonstrated bacterial dissemination in their lung tissues.

**Table 3:** Differentially expressed miRNAs during 8- and 16-weeks of *T. denticola* infection.

Weeks/Infection/Sex	Upregulated miRNAs (*p* < 0.05)	Downregulated miRNAs (*p* < 0.05)
8 Weeks/*T. denticola*-infected vs. 8 Weeks/Sham infection (*n* = 10)	13 (miR-133a, miR-126-5p)	25 (miR-375, miR-34b-5p)
8 Weeks—*T. denticola*-infected Female vs. Male (*n* = 5)	128	14
16 Weeks/*T. denticola*-infected vs. 16 Weeks/Sham infection (*n* = 10)	13 (miR-486, miR-126-3p, miR-126-5p)	19
16 Weeks/*T. denticola*-infected Female vs. Male (*n* = 5)	9	8
8 Weeks/*T. denticola*-infected vs. 16 Weeks/*T. denticola*-infected	9 (miR-126-5p)	19

The number of differentially expressed (DE) miRNAs for *T. denticola*-infected mice after 8- and 16-weeks infection. The commonly expressed miRNAs between 8-weeks and 16-weeks bacterial-infected groups are shown in brackets. Most of the miRNAs expressed in bacterial-infected groups were unique and specific to the 8- and 16-weeks infections.

**Table 4:** Upregulated miRNAs, reported functions, and target genes.

Upregulated miRNAs in 8-Weeks *T. denticola* Infection				
miRs	Fold Change	*p*-Value	Reported Functions	Number of Target Genes
mmu-miR-133a	1.83	0.0176	Abundant in heart, and shown to be involved in the early pathological cascade of myocardial infarction [35].	1426 (e.g., *Med13, Rrp8, Cup, Srrm2, Wipi2, Dlc1, Tgfb2, Rsbn1l, Kat6b, Pold2*)
mmu-miR-378	1.57	0.0198	Major role in hepatic inflammation and fibrosis by targeting NFκB–TNFα axis [36]. Major secreted biomarkers for osteolytic bone metastasis [37]. Expressed to a greater degree in microvesicles of osteoclasts.	--
mmu-miR-22	1.55	0.0012	Promotes cell differentiation, tumor initiation, progression, and metastasis by maintaining Wnt/β-catenin signaling and cancer stem cells function [38]. Observed in the inflammatory mouse lung and brain tissues of polyinosinic-polycytidylic acid-treated mice. Upregulated in periodontal disease and obesity [39].	2150 (e.g., *Arl8b, Syne1, Mbp, Med13, Srrm2, Tpm3, Inpp1, Bin1, Seam3d, Abcg5*)
mmu-miR-136	1.37	0.0066	Potential miRNA biomarker in both experimental and human mild traumatic brain injury [40].	809 (e.g., *Arl8b, Syne1, Med13, Mreg, Rsbn1l, Erc2, Akap8, Sf3a1, Akt2, Apob)*
mmu-miR-2135	1.36	0.0389	Upregulated in chlamydial infection in experimental mice [41].	
mmu-miR-451	1.33	0.0191	Strongly dysregulated in lung cancer and, important miRNA for lung tumor progression [42]. Strongly upregulated in the gingival tissue of periodontitis [43].	
mmu-miR-30a	1.28	0.0254	Overexpressed in gingival tissues in periodontitis patients that inhibits osteogenesis and promotes periodontitis [44].	
mmu-miR-30c	1.25	0.0401	Negative regulator of osteogenic differentiation and bone morphometric protein (BMP)-induced osteogenic differentiation [45].	
mmu-miR-496	1.22	0.0020	Tumor metastasis mediated by Wnt signaling pathways in colorectal cancer [46]. Enforced expression of miR-496 reversed osteogenesis [47].	
mmu-**miR-126-5p**	1.21	0.0148	Major role in ovarian cancer by promoting chemoresistance of ovarian cancer cells [31]. Attenuates blood–brain barrier after ischemic stroke [33].	
mmu-miR-151-5p	1.15	0.0088	Associated with metastasis in breast cancer and hepatocellular carcinoma [48]. Is a therapeutic target for systemic sclerosis [49].	
mmu-miR-720	1.14	0.0111	Promotes glioma growth and upregulates invasion-related genes. Significantly upregulated in glioma tissues and cells [50].	
mmu-miR-100	1.11	0.0484	Overexpressed in hypertrophic hearts, and promotes pathogenesis of cardiac hypertrophy through activation of autophagy [51], a potential protective antiathero-miR [52]. Modulator of cardiac metabolism, ROS production, and protective effect in pressure overload-induced cardiac stress and heart failure [53].	
**Upregulated miRNAs in 16-weeks *T. denticola* Infection**				
mmu-miR-486	1.29	0.0350	Promotes cardiac angiogenesis through fibroblastic MMP19-VEGFA cleavage signaling pathway [54]. Upregulated in chronic myeloid leukemia. Strongly upregulated in the gingival tissue of periodontitis [43].	
mmu-**miR-126-5p**	1.28	0.0050	Major role in ovarian cancer by promoting chemoresistance of ovarian cancer cells [31]. Attenuates blood–brain barrier after ischemic stroke [33]. Promotes the proliferation of endothelial cells and limits atherosclerosis [32].	
mmu-miR-345-5p	1.23	0.0004	Overexpression of miR-345 reduces lipid accumulation in adipocytes and their related genes involved in lipogenic transcription, fatty acid synthesis, and fatty acid transport [55]. Consistently upregulated salivary biomarkers for oral squamous cell carcinoma [56].	124 (e.g., *Dlc1, Rarg, Glu1, Dmgdh, Afmid, Acp2, Inpp5b, Eng, Caprin1, Senp2*)
mmu-miR-132	1.21	0.0100	Inhibits differentiation of periodontal ligament cells into osteoblasts [34].	482 (e.g., *Tthdf3, Wipf, Mapk1, Nek7, Selk, Tspy13, Hus1, Stk35, Arid5b, Plek*)
mmu-**miR-126-3p**	1.2	0.0432	Significantly upregulated in the gingival tissues of chronic and aggressive periodontitis patients [57] and saliva samples of chronic periodontitis [58].	
mmu-miR-101a	1.2	0.0460	Decreased expression observed in serum samples in patients with head and neck squamous cell carcinoma (HNSCC) [59].	
mmu-miR-128	1.18	0.026	Overexpressed in glioma and is a potential noninvasive biomarker to diagnose glioma [60].	
mmu-let-7d	1.17	0.0044	Attenuates epithelial–mesenchymal transition in silica-induced pulmonary fibrosis [61].	
mmu-miR-423-5p	1.17	0.0199	Overexpression of miR-423-5p induces breast cancer cell invasion through NFκB signaling pathway [62].	
mmu-miR-101b	1.17	0.0421	Is a critical mediator of tauopathy and dendritic abnormalities in Alzheimer’s Disease progression [63,64].	
mmu-miR-30b	1.16	0.0453	Overexpressed in osteoarthritis (OA) patients, OA rats, and aggravates joint pain and loss of articular cartilage [65,66].	
mmu-miR-221	1.14	0.0367	Oncogenic miRNA that is involved in many hematologic and solid malignancies, and some nonmalignant diseases [67].	
mmu-miR-26b	1.08	0.0442	Inhibits M1 polarization of microglia by inactivating toll-like receptor pathway [68].	

Details of the target genes were given for the top five significantly expressed miRNAs in both 8-weeks and 16-weeks infected mouse mandibles.

## Data Availability

The data that support the findings of this study are openly available in NCBI at https://www.ncbi.nlm.nih.gov/geo/query/acc.cgi?acc=GSE235373 (accessed on 24 July 2023).

## Supplementary Materials

The following supporting information can be downloaded at: https://www.mdpi.com/article/10.3390/ijms241512105/s1.
