# Supplementary material for: Oral Spirochete Treponema denticola Intraoral Infection Reveals Unique miR-133a, miR-486, miR-126-3p, miR-126-5p miRNA Expression Kinetics during Periodontitis
Source: Int J Mol Sci. 2023 Jul 28;24(15):12105. doi: 10.3390/ijms241512105 (PMC10418472; doi:10.3390/ijms241512105)
Supplement: Supplementary file 1 [file ijms-24-12105-s001.zip › ijms-2525327-SI.pdf]

Oral spirochete *Treponema denticola* intraoral infection reveals unique miR-133a, miR-486, miR-126-3p, miR-126-5p miRNA expression kinetics during periodontitis.

Chairmandurai Aravindraja [1†], Syam Jeepipalli [1], Krishna Mukesh Vekariya [1], Ruben Botello- Escalante [1], Edward K. L. Chan [2] and Lakshmyya Kesavalu [1,2*]

[1] Department of Periodontology, College of Dentistry, University of Florida, Gainesville, FL 32610, USA
[2] Department of Oral Biology, College of Dentistry, University of Florida, Gainesville, FL 32610, USA
* Correspondence: Kesavalu@dental.ufl.edu; Tel.: +1-352-273-6500
† Present address: Department of Neurology, College of Medicine, University of Florida, Gainesville, FL 32610, USA.

Supplemental information

Table S1. Downregulated miRNAs during 8-weeks of infection, molecular functions, and target genes.

miRNAs	Fold change	*p*-value	Reported functions	Number of Target Genes
miR-375	-2.02	0.0421	Down regulated in oral squamous cell carcinoma [1]. Salivary adenoid cystic carcinoma had downregulated miR-375 [2].	276 *(Bmpr2, Mtpn, Klf4, Enah, Fzd8, F10, Zfpm2, Atxn7, Rs1, Tsc1, Pdpk1, Uvssa, Nufip2)*
miR-34b-5p	-1.73	0.0357	Enhance the resistance to bleomycin by regulating its target gene TIMP3 during the pathogenesis of lung fibrosis [3].	355 *(Arhgap1, Ppp4r2, Dixdc1, Htr2c, Rpap1, N4bp1, Tbc1d2b, Rab3c)*
miR-210	-1.55	0.0095	Induces endothelial dysfunction in type 2 diabetes. Promoting regulatory T-cell signaling in periodontitis [4].	16 *(Onecut3, Chst4, Sept8, Gramd1c, Ccdc150)*
miR-1902	-1.54	0.0206	Distinct microRNA expression profiles in mouse renal cortical tissue [5].	92 *(Tsc1, Spryd3, Lrtm1, Ndufs4, Calm1)*
miR-203	-1.45	0.0026	Lower in imatinib-resistant GBM cells [6]. Downregulated in colorectal cancer tissues [7].	483 *(Sema3d, Lyzl1, Kat6b, Col22a1, Fbxo33)*
miR-376a	-1.44	0.0077	Downregulated in gastric cancer tissue [8].	06

miR-let-7a	-1.37	7.04E-05	Let-7a-5p was revealed to be downregulated by 21.67% in pneumoconiosis [9]. Downregulation promoted insulin receptor /insulin-like growth factor signaling in pancreatic ductal adenocarcinoma [10].	541
miR-148a	-1.34	0.0346	Downregulated in hypoxia condition of human colorectal cancer cell lines [11].	331
miR-362-3p	-1.33	0.0019	Aberrant miR-362-3p is associated with EBV-infection and prognosis in Nasopharyngeal Carcinoma [12].	883
miR-574-5p	-1.33	0.0182	Decreasing the HK-2 cells viability in acute kidney injury patients [13].	424
miR-134	-1.32	0.0178	Significantly downregulated in major depressive disorder [14].	173
miR-882	-1.31	0.0390	Osteoclastogenesis models has downregulated levels of miR-882 [15].	218
miR-678	-1.3	0.0113	No function reported.	6
mcmv-miR-m108-1	-1.29	0.0340	No function reported.	--
miR-423-5p	-1.28	0.0203	Effectively regulate the radiosensitivity in colorectal cancer [16].	216
miR-883a-3p	-1.27	0.0399	No function reported	273
miR-202-5p	-1.27	0.0493	Downregulated levels have a role in myocardial ischemia [17].	155
miR-let-7f	-1.26	0.0004	Early stages of epithelial ovarian cancer tissue and serum samples had reduced levels [18].	529
miR-187	-1.26	0.0298	Gastric cancer condition has downregulated levels of miR-187 [19].	21
miR-let-7c	-1.25	0.0036	Downregulation had a reported role in pathogenesis of human hepatocellular carcinoma [20].	558

miR-467e	-1.23	0.0483		83
miR-29b	-1.22	0.0048	Fibrous plaques of Peyronie's disease has downregulated expression [21].	452
miR-23a	-1.17	0.0186	Regulating the functions of inflammatory response in the septic insult condition [22].	546
miR-98	-1.14	0.0181	Reported functions of tumor development in pancreatic ductal adenocarcinoma [23].	541
miR-let-7e	-1.1	0.0155	Downregulated levels observed in the cerebral palsy condition [24].	538

Table S2. Downregulated miRNAs during 16-weeks infection, molecular functions, and target genes.

**miRNAs**	**Fold change**	***p*-value**	**Reported functions**	**Number of Target Genes**
miR-2135	-1.48	3.61E-05	Downregulated in the analgesic and addictive drug – morphine addiction [25].	--
miR-142-3p	-1.39	0.0226	Enhanced IFN-γ expression observed in downregulated levels [26].	215 *(Dcakd, Rarg, Atg4c, Tbc1d2b, Fkbp1a)*
miR-130a	-1.25	0.0067	Oral squamous cell carcinoma can be controlled by downregulated levels [27].	366 *(Zfp113, Slain1, Apcdd1, Sos2, Mtf1)*
miR-720	-1.24	0.0047	Promoting insulin secretion in MIN6 cells [28].	--
miR-1274a	-1.22	0.0137	Patients with colon cancer have increased survival rate on downregulated miR-1274a [29].	No target gene detected
miR-10a	-1.22	0.0236	Downregulated in the wound healing process [30].	178 *(Nup50, Nr6a1, Epha5, Sobp, Wipf1)*
miR-151-3p	-1.21	2.87E-05	Mice with cardiac hypertrophy have downregulated levels of miR-151-3p [31].	57
miR-496	-1.21	0.0107	Gastric cancer cells of AGS and MKN45 has downregulated levels of miR-496 which affects AKT/mTOR signaling pathway [32].	113

miR-125b-5p	-1.2	0.0121	Small extracellular vesicles present in aging related diseases has downregulated expression of miR-125-5p levels [33].	496
miR-323-3p	-1.19	0.0169	Significant downregulation observed in Pancreatic ductal adenocarcinoma tissue and cell lines [34].	356
miR-195	-1.18	0.0176	Downregulated levels in hepatocellular carcinoma [35].	583
miR-582-5p	-1.16	0.0174	Bladder cancer cells and tissue has decreased expression [36].	396
miR-1944	-1.16	0.0176	Observed in cognitive impairment condition of Alzheimer's disease [37].	--
miR-106b	-1.16	0.0179	Attenuating the inflammation response in collagen-induced arthritis [38] and downregulated in mycobacterial infection [39].	761
miR-324-5p	-1.15	0.0173	Oxygen glucose deprivation mediated neurological injury has downregulated levels [40].	19
miR-15a	-1.11	0.0348	Downregulated levels observed in Apelin-mediated lung cancer development [41].	--
miR-16	-1.11	0.0465	Decreased the apoptotic rate in hypoxia/reoxygenation damage [42].	--
miR-25	-1.1	0.0275	Downregulated in the inflammed tracheal smooth muscle cells [43].	
miR-107	-1.1	0.0449	Downregulated miR-107 expression was associated with advanced Ann Arbor stage, high IPI score, LDH, and β2-MG level in DLBCL patients [13].	369

Table S3. Comparison of downregulated miRNAs between 8-weeks and 16-weeks of infection, molecular functions, and target genes.

miRNAs	Fold change	*p*-value	Reported functions	Number of Target Genes
miR-2135	-1.77	0.0001	Downregulated in the analgesic and addictive drug – morphine [25].	No target gene detected
miR-2133	-1.58	0.0024	Downregulated in macrophage transition of M0 to M2a in microglia [44].	No target gene detected
miR-223	-1.57	0.0159	*Streptococcus* inflammation in murine macrophages decreased miR-223 levels [45].	222 *(Orc4, Armcx1, Gpr155, Zbtb18, Pknox1)*
miR-142-3p	-1.44	0.0299	Enhanced IFN-γ expression observed under downregulated levels [26].	*215* (*Dcakd, Rarg, Atg4c, Tbc1d2b, Fkbp1a)*
miR-130a	-1.35	0.0006	Oral squamous cell carcinoma can be controlled by downregulated levels [27].	366 (*Zfp113, Slain1, Apcdd1, Sos2, Mtf1)*
miR-342-3p	-1.27	0.0077	Downregulated in Hepatocellular carcinoma condition [46].	309 (*Apmap, Prkaa2, Trim39, Sla, Pde6h*)
miR-496	-1.26	0.0006	Gastric cancer cells of AGS and MKN45 has downregulated levels of miR-496 which affecting AKT/mTOR signaling pathway [32].	113
miR-150	-1.25	0.0088	Myasthenia gravis condition has downregulated levels [47].	248
miR-720	-1.22	0.0019	Promoting insulin secretion in MIN6 cells [28].	--
miR-125b-5p	-1.22	0.0043	Small extracellular vesicles present in aging related diseases has downregulated expression of miR-125-5p levels [33].	496
miR-451	-1.21	0.0382	Downregulated in Nasopharyngeal carcinoma [48].	17
miR-15a	-1.18	0.01	Downregulated levels observed in Apelin- mediated lung cancer development [41].	--
miR-338-3p	-1.17	0.0492	Decreased levels observed in LO2 cells of hepatocellular carcinoma [49].	319
miR-1944	-1.16	0.0239	Observed in cognitive impairment condition of Alzheimer's disease [37].	--

miR-99a	-1.15	0.0313	Downregulation of miRNA 99a in oral squamous cell carcinomas contributes to the growth and survival of oral cancer cells [50].	15
miR-25	-1.13	0.0019	Downregulated in the inflamed tracheal smooth muscle cells [43].	369
miR-151-3p	-1.13	0.0092	Mice with cardiac hypertrophy demonstrated downregulated levels of miR-151-3p [31].	57
miR-324-5p	-1.07	0.0466	Oxygen glucose deprivation mediated neurological injury has downregulated levels [40].	19

Table S4. miRTarBase analysis of upregulated DE microRNAs and their target genes in 8 weeks *T. denticola* infection.

miRTarBase ID	miRNA	Target gene
MIRT000604	mmu-miR-133a-3p	*Nfatc4*
MIRT001959	mmu-miR-133a-3p	*Runx2*
MIRT002895	mmu-miR-133a-3p	*Cdc42*
MIRT002896	mmu-miR-133a-3p	*Whsc2*
MIRT002897	mmu-miR-133a-3p	*Rhoa*
MIRT004022	mmu-miR-133a-3p	*Ucp2*
MIRT004102	mmu-miR-133a-3p	*Casp9*
MIRT004267	mmu-miR-133a-3p	*Spry1*
MIRT004652	mmu-miR-133a-3p	*Ccnd2*
MIRT004653	mmu-miR-133a-3p	*Srf*
MIRT004833	mmu-miR-133a-3p	*Hdac4*
MIRT005400	mmu-miR-133a-3p	*Pola1*
MIRT006261	mmu-miR-133a-3p	*Igf1r*
MIRT015113	mmu-miR-133a-5p	*Rhoa*
MIRT053603	mmu-miR-133a-3p	*Prdm16*
MIRT054781	mmu-miR-133a-3p	*Kcnmb1*
MIRT438724	mmu-miR-133a-3p	*Rapgef3*
MIRT438725	mmu-miR-133a-3p	*Prkacb*
MIRT438726	mmu-miR-133a-3p	*Adcy6*
MIRT438727	mmu-miR-133a-3p	*Adrb1*
MIRT593588	mmu-miR-133a-3p	*Zfp26*
MIRT594422	mmu-miR-133a-3p	*Gdf3*
MIRT599889	mmu-miR-133a-3p	*Acer1*
MIRT604592	mmu-miR-133a-3p	*Tnrc6b*
MIRT743586	mmu-miR-133a	*Acer1*
MIRT746521	mmu-miR-133a	*Tnrc6b*
MIRT748596	mmu-miR-133a	*Zfp26*
MIRT749511	mmu-miR-133a	*Gdf3*

We used mmu-miR-133 an example for an upregulated DE miRNA during 8 weeks of infection in identifying the target genes using the miRTarBase. Each miRNA has different target genes and each with a specific MiRTarBase ID. *T. denticola*-infection induced DE upregulated mmu-miR-133 has 28 different target genes with 28 different MiRTarBase ID as stated in the table.

Table S5. miRTarBase analysis of upregulated DE microRNAs and their target genes in 16 weeks *T. denticola* infection.

**miRTarBase ID**	**miRNA**	**Target gene**
MIRT744264	mmu-miR-486	*H2-Q4*
MIRT747084	mmu-miR-486	*Maoa*
MIRT750467	mmu-miR-486	*Zcchc9*
MIRT744588	mmu-miR-486	*Ms4a6c*
MIRT746127	mmu-miR-486	*Cnnm3*
MIRT747348	mmu-miR-486	*Tbc1d8b*
MIRT744650	mmu-miR-486	*Nkain3*
MIRT742609	mmu-miR-486	*Shc4*
MIRT749957	mmu-miR-486	*Tns3*
MIRT745593	mmu-miR-486	*Aim2*
MIRT748160	mmu-miR-486	*Gm7609*
MIRT753322	mmu-miR-486	*Fam129c*
MIRT598720	mmu-miR-486a-5p	*H2-Q4*
MIRT598358	mmu-miR-486a-5p	*Maoa*
MIRT590827	mmu-miR-486a-5p	*Zcchc9*
MIRT606321	mmu-miR-486a-5p	*Ms4a6c*
MIRT601041	mmu-miR-486a-5p	*Cnnm3*
MIRT597071	mmu-miR-486a-5p	*Tbc1d8b*
MIRT598042	mmu-miR-486a-5p	*Nkain3*
MIRT581056	mmu-miR-486a-5p	*Shc4*
MIRT592961	mmu-miR-486a-5p	*Tns3*
MIRT599803	mmu-miR-486a-5p	*Aim2*
MIRT598800	mmu-miR-486a-5p	*Gm7609*
MIRT578874	mmu-miR-486a-5p	*Fam129c*
MIRT003791	mmu-miR-486a-5p	*Pax7*
MIRT003527	mmu-miR-486a-5p	*Pten*
MIRT004586	mmu-miR-486a-5p	*Foxo1*
MIRT736452	mmu-miR-486a-5p	*Igf2bp3*
MIRT598721	mmu-miR-486b-5p	*H2-Q4*
MIRT598359	mmu-miR-486b-5p	*Maoa*
MIRT590828	mmu-miR-486b-5p	*Zcchc9*

MIRT606320	mmu-miR-486b-5p	*Ms4a6c*
MIRT601040	mmu-miR-486b-5p	*Cnnm3*
MIRT597070	mmu-miR-486b-5p	*Tbc1d8b*
MIRT598043	mmu-miR-486b-5p	*Nkain3*
MIRT581057	mmu-miR-486b-5p	*Shc4*
MIRT592960	mmu-miR-486b-5p	*Tns3*
MIRT599804	mmu-miR-486b-5p	*Aim2*
MIRT598799	mmu-miR-486b-5p	*Gm7609*
MIRT578873	mmu-miR-486b-5p	*Fam129c*
MIRT736777	mmu-miR-486b-3p	*Pten*

We used mmu-miR-486 an example for an upregulated DE miRNA during 16 weeks of infection in identifying the target genes using the miRTarBase. Each miRNA has different target genes and each with a specific MiRTarBase ID. *T. denticola*-infection induced DE upregulated mmu-miR-486 has 41 different target genes with 41 different MiRTarBase ID as stated in the table.

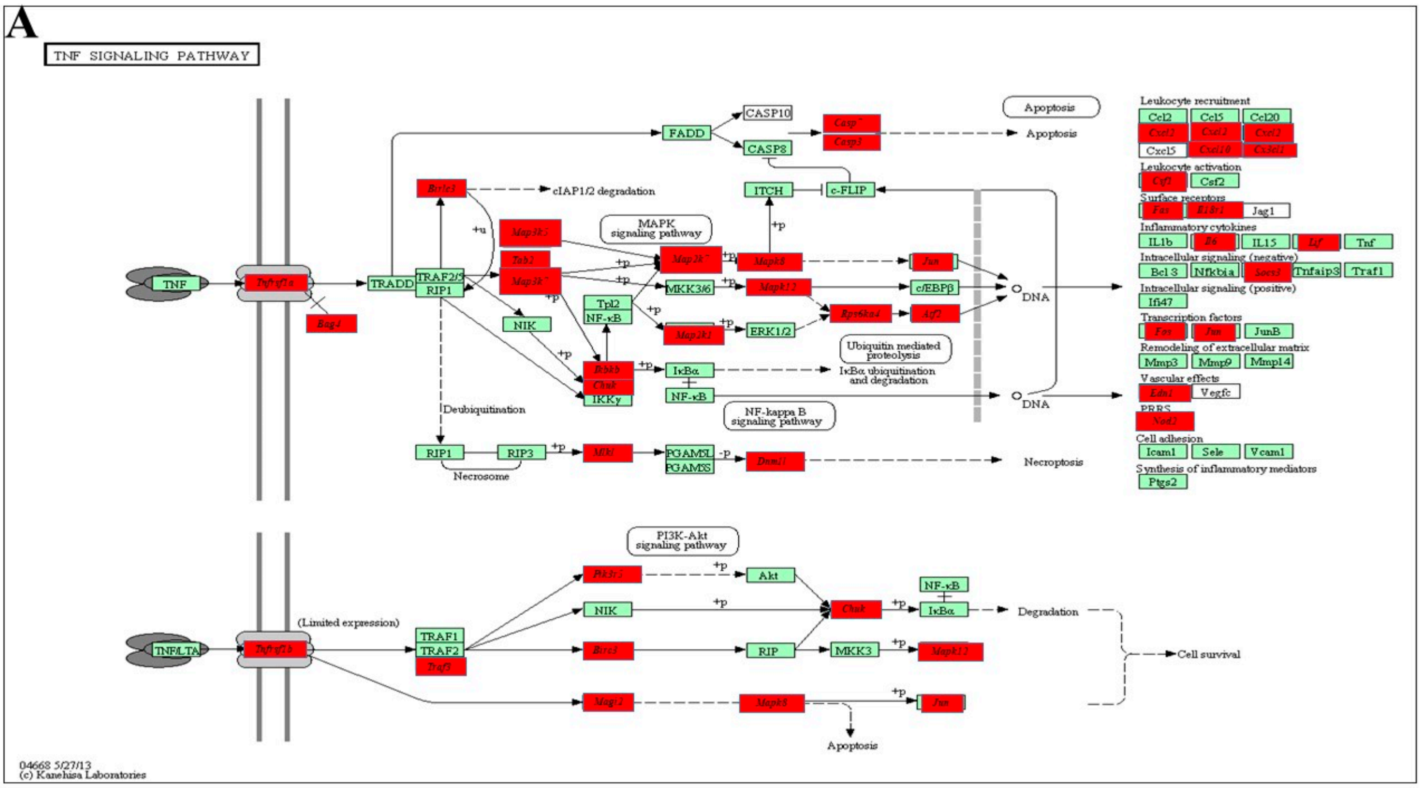

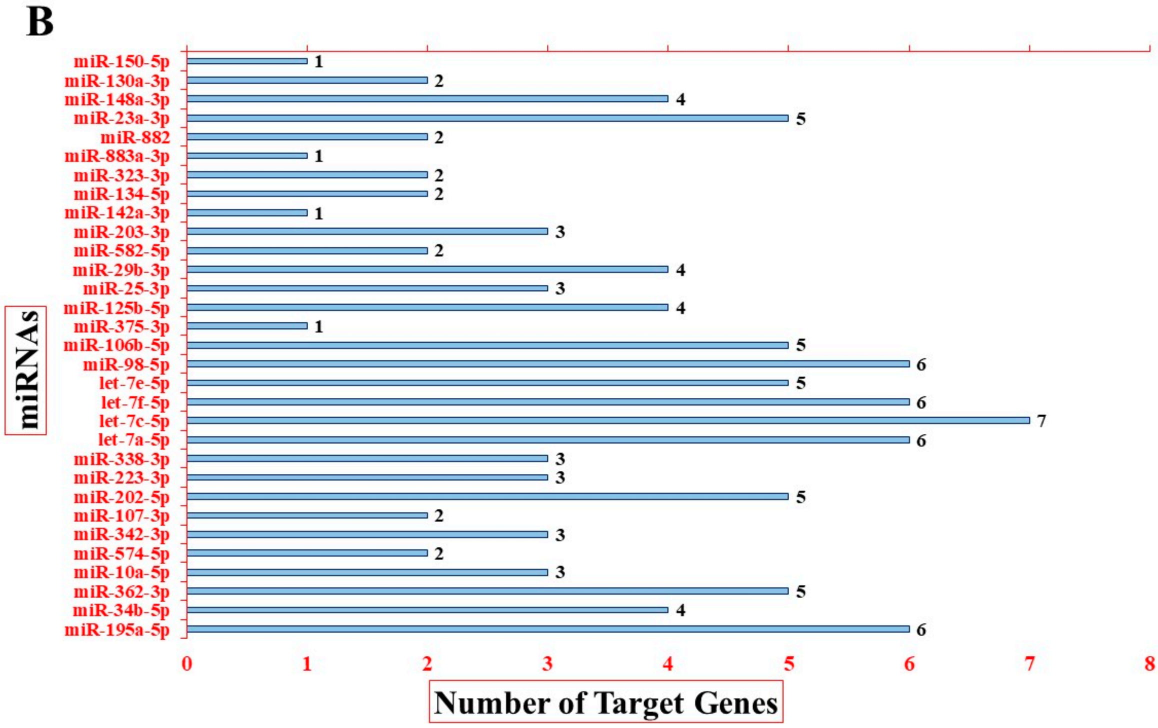

Figure S1. Significant differentially expressed (DE) genes (identified by KEGG) involved in the TNF signaling pathway (A). Red boxes indicate significantly altered expression of genes (e.g. leucocyte recruitment genes Cxcl2, Cxcl10; leucocyte activation gene Csf1; inflammatory cytokine genes IL-6, lif) based on *T. denticola*-induced miRNA profiles from Nanostring analysis. List of miRNAs and their target genes associated with TNF signaling pathway (B).

References

1. Siow, M.Y.; Ng, L.P.; Vincent-Chong, V.K.; Jamaludin, M.; Abraham, M.T.; Abdul Rahman, Z.A.; Kallarakkal, T.G.; Yang, Y.H.; Cheong, S.C.; Zain, R.B. Dysregulation of miR-31 and miR-375 expression is associated with clinical outcomes in oral carcinoma. *Oral Dis* **2014**, *20*, 345-351, doi:10.1111/odi.12118.
2. Mitani, Y.; Roberts, D.B.; Fatani, H.; Weber, R.S.; Kies, M.S.; Lippman, S.M.; El-Naggar, A.K. MicroRNA profiling of salivary adenoid cystic carcinoma: association of miR-17-92 upregulation with poor outcome. *PLoS One* **2013**, *8*, e66778, doi:10.1371/journal.pone.0066778.
3. Lu, Y.Y.; Zhang, X.J. MiR-34b-5p knockdown attenuates bleomycin-induced pulmonary fibrosis by targeting tissue inhibitor of metalloproteinase 3 (TIMP3). *Rivista europea per le scienze mediche e farmacologiche = European review for medical and pharmacological sciences.* **2019**, *23*, 2273-2279, doi:10.26355/eurrev_201903_17276.
4. Parachuru, V.P.; Coates, D.E.; Milne, T.J.; Hussaini, H.M.; Rich, A.M.; Seymour, G.J. Forkhead box P3-positive regulatory T-cells and interleukin 17-positive T-helper 17 cells in chronic inflammatory periodontal disease. *J Periodontal Res* **2014**, *49*, 817-826, doi:10.1111/jre.12169.
5. Schuler, E.; Parris, T.Z.; Helou, K.; Forssell-Aronsson, E. Distinct microRNA expression profiles in mouse renal cortical tissue after 177Lu-octreotate administration. *PLoS One* **2014**, *9*, e112645, doi:10.1371/journal.pone.0112645.
6. Liao, H.Z.; Bai, Y.F.; Qiu, S.C.; Zheng, L.; Huang, L.Y.; Liu, T.Z.; Wang, X.; Liu, Y.T.; Xu, N.B.; Yan, X.H.; et al. MiR-203 downregulation is responsible for chemoresistance in human glioblastoma by promoting epithelial-mesenchymal transition via SNAI2. *Oncotarget* **2015**, *6*, 8914-8928, doi:DOI 10.18632/oncotarget.3563.
7. Deng, B.; Wang, B.; Fang, J.; Zhu, X.; Cao, Z.; Lin, Q.; Zhou, L.; Sun, X. MiRNA-203 suppresses cell proliferation, migration and invasion in colorectal cancer via targeting of EIF5A2. *Sci Rep* **2016**, *6*, 28301, doi:10.1038/srep28301.
8. Zhang, C.; Liang, Y.; Ma, M.H.; Wu, K.Z.; Zhang, C.D.; Dai, D.Q. Downregulation of microRNA-376a in Gastric Cancer and Association with Poor Prognosis. *Cell Physiol Biochem* **2018**, *51*, 2010-2018, doi:10.1159/000495820.
9. Zhang, L.; Hao, C.; Zhai, R.; Wang, D.; Zhang, J.; Bao, L.; Li, Y.; Yao, W. Downregulation of exosomal let-7a-5p in dust exposed- workers contributes to lung cancer development. *Respir Res* **2018**, *19*, 235, doi:10.1186/s12931-018-0949-y.
10. Nweke, E.E.; Brand, M. Downregulation of the let-7 family of microRNAs may promote insulin receptor/insulin-like growth factor signalling pathways in pancreatic ductal adenocarcinoma. *Oncol Lett* **2020**, *20*, 2613-2620, doi:10.3892/ol.2020.11854.
11. Nersisyan, S.; Galatenko, A.; Chekova, M.; Tonevitsky, A. Hypoxia-Induced miR-148a Downregulation Contributes to Poor Survival in Colorectal Cancer. *Front Genet* **2021**, *12*, 662468, doi:10.3389/fgene.2021.662468.
12. Wang, X.Y.; Chen, P. Aberrant miR-362-3p is Associated with EBV-Infection and Prognosis in Nasopharyngeal Carcinoma and Involved in Tumor Progression by Targeting JMJD2A. *Oncotargets and Therapy* **2022**, *15*, 121-131, doi:10.2147/Ott.S325100.
13. Liu, S.; Zhao, L.; Zhang, L.; Qiao, L.; Gao, S. Downregulation of miR-574-5p inhibits HK-2 cell viability and predicts the onset of acute kidney injury in sepsis patients. *Ren Fail* **2021**, *43*, 942-948, doi:10.1080/0886022X.2021.1939051.

14. Zhang, H.P.; Liu, X.L.; Chen, J.J.; Cheng, K.; Bai, S.J.; Zheng, P.; Zhou, C.J.; Wang, W.; Wang, H.Y.; Zhong, L.M.; et al. Circulating microRNA 134 sheds light on the diagnosis of major depressive disorder. *Transl Psychiatry* **2020**, *10*, 95, doi:10.1038/s41398-020-0773-2.
15. Tian, Y.; Gong, Z.; Zhao, R.; Zhu, Y. Melatonin inhibits RANKL-induced osteoclastogenesis through the miR-882/Rev-erbα axis in Raw264.7 cells. *Int J Mol Med* **2021**, *47*, 633-642, doi:10.3892/ijmm.2020.4820.
16. Shang, Y.; Wang, L.; Zhu, Z.; Gao, W.; Li, D.; Zhou, Z.; Chen, L.; Fu, C.G. Downregulation of miR-423-5p Contributes to the Radioresistance in Colorectal Cancer Cells. *Front Oncol* **2020**, *10*, 582239, doi:10.3389/fonc.2020.582239.
17. Li, Y.; Li, Q.; Zhang, O.; Guan, X.; Xue, Y.; Li, S.; Zhuang, X.; Zhou, B.; Miao, G. miR-202-5p protects rat against myocardial ischemia reperfusion injury by downregulating the expression of Trpv2 to attenuate the Ca (2+) overload in cardiomyocytes. *J Cell Biochem* **2019**, *120*, 13680-13693, doi:10.1002/jcb.28641.
18. Kumar, V.; Gupta, S.; Varma, K.; Chaurasia, A.; Sachan, M. Diagnostic performance of microRNA-34a, let-7f and microRNA-31 in epithelial ovarian cancer prediction. *J Gynecol Oncol* **2022**, *33*, e49, doi:10.3802/jgo.2022.33.e49.
19. Chen, W.; Cui, Y.; Wang, J.; Yuan, Y.; Sun, X.; Zhang, L.; Shen, S.; Cheng, J. Effects of downregulated expression of microRNA-187 in gastric cancer. *Exp Ther Med* **2018**, *16*, 1061-1070, doi:10.3892/etm.2018.6318.
20. Zhu, X.; Wu, L.; Yao, J.; Jiang, H.; Wang, Q.; Yang, Z.; Wu, F. MicroRNA let-7c Inhibits Cell Proliferation and Induces Cell Cycle Arrest by Targeting CDC25A in Human Hepatocellular Carcinoma. *PLoS One* **2015**, *10*, e0124266, doi:10.1371/journal.pone.0124266.
21. Dos Santos, V.G.; Dos Santos, G.A.; Neto, C.B.; Viana, N.I.; Pimenta, R.; Guimarães, V.R.; Candido, P.; Romão, P.; de Camargo, J.A.; Leite, K.R.M.; et al. Downregulation of miR-29b is associated with Peyronie's disease. *Urologia* **2022**, *89*, 451-455, doi:10.1177/03915603211036628.
22. Si, X.; Cao, D.; Chen, J.; Nie, Y.; Jiang, Z.; Chen, M.Y.; Wu, J.F.; Guan, X.D. miR-23a downregulation modulates the inflammatory response by targeting ATG12-mediated autophagy. *Mol Med Rep* **2018**, *18*, 1524-1530, doi:10.3892/mmr.2018.9081.
23. Fu, Y.; Liu, X.; Chen, Q.; Liu, T.; Lu, C.; Yu, J.; Miao, Y.; Wei, J. Downregulated miR-98-5p promotes PDAC proliferation and metastasis by reversely regulating MAP4K4. *J Exp Clin Cancer Res* **2018**, *37*, 130, doi:10.1186/s13046-018-0807-2.
24. Vechetti, I.J.; Norrbom, J.; Alkner, B.; Hjalmarsson, E.; Palmcrantz, A.; Pontén, E.; Pingel, J.; von Walden, F.; Fernandez-Gonzalo, R. Extracellular vesicle characteristics and microRNA content in cerebral palsy and typically developed individuals at rest and in response to aerobic exercise. *Front Physiol* **2022**, *13*, 1072040, doi:10.3389/fphys.2022.1072040.
25. Wang, T.; Chen, X.; Zeng, K. Molecular mechanism and candidate biomarkers of morphine for analgesia and addiction effects. *Ann Transl Med* **2022**, *10*, 89, doi:10.21037/atm-21-7037.
26. Ma, N.; Wei, T.; Wang, B.; Jiang, X.; Zhou, L.; Zhong, R. MicroRNA-142-3p inhibits IFN-γ production via targeting of RICTOR in. *Ann Transl Med* **2019**, *7*, 649, doi:10.21037/atm.2019.10.85.
27. Peng, Y.; Hu, S.; Zhang, K.; Wang, Y.; Rouzi, M.; Zhou, D.; Yang, R. Downregulation of MicroRNA-130a Inhibits Oral Squamous Cell Carcinoma Proliferation and Metastasis via the Hippo-YAP Pathway. *Cancer Manag Res* **2021**, *13*, 4829-4840, doi:10.2147/CMAR.S287575.

28. Lu, C.; Wang, D.; Feng, Y.; Feng, L.; Li, Z. miR-720 Regulates Insulin Secretion by Targeting Rab35. *Biomed Res Int* **2021**, *2021*, 6662612, doi:10.1155/2021/6662612.
29. Ren, B.; Yang, B.; Li, P.; Ge, L. Upregulation of MiR-1274a is Correlated with Survival Outcomes and Promotes Cell Proliferation, Migration, and Invasion of Colon Cancer. *Onco Targets Ther* **2020**, *13*, 6957-6966, doi:10.2147/OTT.S246160.
30. Simões, A.; Chen, L.; Chen, Z.; Zhao, Y.; Gao, S.; Marucha, P.T.; Dai, Y.; DiPietro, L.A.; Zhou, X. Differential microRNA profile underlies the divergent healing responses in skin and oral mucosal wounds. *Sci Rep* **2019**, *9*, 7160, doi:10.1038/s41598-019-43682-w.
31. Wei, H.; Li, Z.; Wang, X.; Wang, J.; Pang, W.; Yang, G.; Shen, Q.W. microRNA-151-3p regulates slow muscle gene expression by targeting ATP2a2 in skeletal muscle cells. *J Cell Physiol* **2015**, *230*, 1003-1012, doi:10.1002/jcp.24793.
32. Su, R.; Zhao, E.; Zhang, J. miR-496 inhibits proliferation via LYN and AKT pathway in gastric cancer. *Open Med (Wars)* **2021**, *16*, 1206-1214, doi:10.1515/med-2021-0313.
33. Kim, C.G.; Lee, J.K.; Cho, G.J.; Shin, O.S.; Gim, J.A. Small RNA sequencing of small extracellular vesicles secreted by umbilical cord mesenchymal stem cells following replicative senescence. *Genes Genomics* **2023**, *45*, 347-358, doi:10.1007/s13258-022-01297-y.
34. Wang, C.; Liu, P.; Wu, H.; Cui, P.; Li, Y.; Liu, Y.; Liu, Z.; Gou, S. MicroRNA-323-3p inhibits cell invasion and metastasis in pancreatic ductal adenocarcinoma via direct suppression of SMAD2 and SMAD3. *Oncotarget* **2016**, *7*, 14912-14924, doi:10.18632/oncotarget.7482.
35. Yu, S.; Jing, L.; Yin, X.R.; Wang, M.C.; Chen, Y.M.; Guo, Y.; Nan, K.J.; Han, L.L. MiR-195 suppresses the metastasis and epithelial-mesenchymal transition of hepatocellular carcinoma by inhibiting YAP. *Oncotarget* **2017**, *8*, 99757-99771, doi:10.18632/oncotarget.20909.
36. Tian, Y.; Guan, Y.; Su, Y.; Luo, W.; Yang, G.; Zhang, Y. MiR-582-5p Inhibits Bladder Cancer-Genesis by Suppressing TTK Expression. *Cancer Manag Res* **2020**, *12*, 11933-11944, doi:10.2147/CMAR.S274835.
37. Chum, P.P.; Hakim, M.A.; Behringer, E.J. Cerebrovascular microRNA Expression Profile During Early Development of Alzheimer's Disease in a Mouse Model. *J Alzheimers Dis* **2022**, *85*, 91-113, doi:10.3233/JAD-215223.
38. Tao, Y.; Wang, Z.; Wang, L.; Shi, J.; Guo, X.; Zhou, W.; Wu, X.; Liu, Y.; Zhang, W.; Yang, H.; et al. Downregulation of miR-106b attenuates inflammatory responses and joint damage in collagen-induced arthritis. *Rheumatology (Oxford)* **2017**, *56*, 1804-1813, doi:10.1093/rheumatology/kex233.
39. Pires, D.; Bernard, E.M.; Pombo, J.P.; Carmo, N.; Fialho, C.; Gutierrez, M.G.; Bettencourt, P.; Anes, E. Modulates miR-106b-5p to Control Cathepsin S Expression Resulting in Higher Pathogen Survival and Poor T-Cell Activation. *Front Immunol* **2017**, *8*, 1819, doi:10.3389/fimmu.2017.01819.
40. Gu, J.; Gui, S.; Hu, L.; Kong, L.; Di, M.; Wang, Y. Downregulated miRNA-324-5p aggravates neuronal injury induced by oxygen-glucose deprivation via modulating RAN. *Exp Ther Med* **2020**, *19*, 658-664, doi:10.3892/etm.2019.8249.
41. Ran, J.; Li, Y.; Liu, L.; Zhu, Y.; Ni, Y.; Huang, H.; Liu, Z.; Miao, Z.; Zhang, L. Apelin enhances biological functions in lung cancer A549 cells by downregulating exosomal miR-15a-5p. *Carcinogenesis* **2021**, *42*, 243-253, doi:10.1093/carcin/bgaa089.
42. Zhang, H.J.; Zhang, Y.N.; Teng, Z.Y. Downregulation of miR-16 protects H9c2(2-1) cells against hypoxia/reoxygenation damage by targeting CIAPIN1 and regulating the NF-κB pathway. *Mol Med Rep* **2019**, *20*, 3113-3122, doi:10.3892/mmr.2019.10568.
43. Sárközy, M.; Kahán, Z.; Csont, T. A myriad of roles of miR-25 in health and disease. *Oncotarget* **2018**, *9*, 21580-21612, doi:10.18632/oncotarget.24662.

44. Freilich, R.W.; Woodbury, M.E.; Ikezu, T. Integrated expression profiles of mRNA and miRNA in polarized primary murine microglia. *PLoS One* **2013**, *8*, e79416, doi:10.1371/journal.pone.0079416.
45. Li, G.; Zong, X.; Cheng, Y.; Xu, J.; Deng, J.; Huang, Y.; Ma, C.; Fu, Q. miR-223-3p contributes to suppressing NLRP3 inflammasome activation in *Streptococcus equi* ssp. zooepidemicus infection. *Vet Microbiol* **2022**, *269*, 109430, doi:10.1016/j.vetmic.2022.109430.
46. Komoll, R.M.; Hu, Q.; Olarewaju, O.; von Döhlen, L.; Yuan, Q.; Xie, Y.; Tsay, H.C.; Daon, J.; Qin, R.; Manns, M.P.; et al. MicroRNA-342-3p is a potent tumour suppressor in hepatocellular carcinoma. *J Hepatol* **2021**, *74*, 122-134, doi:10.1016/j.jhep.2020.07.039.
47. Cron, M.A.; Maillard, S.; Truffault, F.; Gualeni, A.V.; Gloghini, A.; Fadel, E.; Guihaire, J.; Behin, A.; Berrih-Aknin, S.; Le Panse, R. Causes and Consequences of miR-150-5p Dysregulation in Myasthenia Gravis. *Front Immunol* **2019**, *10*, 539, doi:10.3389/fimmu.2019.00539.
48. Liu, N.; Jiang, N.; Guo, R.; Jiang, W.; He, Q.M.; Xu, Y.F.; Li, Y.Q.; Tang, L.L.; Mao, Y.P.; Sun, Y.; et al. MiR-451 inhibits cell growth and invasion by targeting MIF and is associated with survival in nasopharyngeal carcinoma. *Mol Cancer* **2013**, *12*, 123, doi:10.1186/1476-4598-12-123.
49. Fu, X.; Tan, D.; Hou, Z.; Hu, Z.; Liu, G. miR-338-3p is down-regulated by hepatitis B virus X and inhibits cell proliferation by targeting the 3'-UTR region of CyclinD1. *Int J Mol Sci* **2012**, *13*, 8514-8539, doi:10.3390/ijms13078514.
50. Yan, B.; Fu, Q.; Lai, L.; Tao, X.; Fei, Y.; Shen, J.; Chen, Z.; Wang, Q. Downregulation of microRNA 99a in oral squamous cell carcinomas contributes to the growth and survival of oral cancer cells. *Mol Med Rep* **2012**, *6*, 675-681, doi:10.3892/mmr.2012.971.
